# Exploring reasons for MD-PhD trainees’ experiences of impostor phenomenon

**DOI:** 10.1186/s12909-022-03396-6

**Published:** 2022-04-30

**Authors:** Devasmita Chakraverty, Jose E. Cavazos, Donna B. Jeffe

**Affiliations:** 1 Ravi J. Matthai Centre for Educational Innovation, India Institute of Management Ahmedabad, KLMDC# 36, Old Campus, Ahmedabad, Gujarat 380 015 India; 2 South Texas Medical Scientist Training Program, University of Texas Health San Antonio, San Antonio, TX 78229 USA; 3 Washington University School of Medicine, St. Louis, MO 63110 USA

**Keywords:** Medical education, MD-PhD training, Physician-scientist, Impostor phenomenon, bias and micro-aggressions, Clinical-scientist, Professional identity formation

## Abstract

**Background:**

Acceptance into U.S. MD-PhD dual-degree programs is highly competitive, and the lengthy training program requires transitioning between multiple phases (pre-clinical-, PhD-research-, and clinical-training phases), which can be stressful. Challenges faced during MD-PhD training could exacerbate self-doubt and anxiety. Impostor phenomenon is the experience of feeling like a fraud, with some high-achieving, competent individuals attributing their successes to luck or other factors rather than their own ability and hard work. To our knowledge, impostor phenomenon among MD-PhD trainees has not been described. This study examined impostor phenomenon experiences during MD-PhD training and reasons trainees attributed to these feelings.

**Methods:**

Individuals in science and medicine fields participated in an online survey that included the 20-item Clance Impostor Phenomenon Scale (CIPS); higher scores (range 20–100) indicate more frequent impostor phenomenon. Some respondents who reported experiencing impostor phenomenon also voluntarily completed a semi-structured interview, sharing experiences during training that contributed to feelings of impostor phenomenon. Interview transcripts were coded and analysed using the constant comparative method and analytic induction to identify themes.

**Results:**

Of 959 survey respondents (students and professionals in science and medicine), 13 MD-PhD students and residents completed the survey, nine of whom (five male, four female; four white, five other race-ethnicity) also completed an interview. These participants experienced moderate-to-intense scores on the CIPS (range: 46–96). Four themes emerged from the interview narratives that described participants’ experiences of IP: professional identity formation, fear of evaluation, minority status, and, program-transition experiences. All reported struggling to develop a physician-scientist identity and lacking a sense of belonging in medicine or research.

**Conclusions:**

Impostor experiences that MD-PhD participants attributed to bias and micro-aggressions in social interactions with peers, faculty, and patients challenged their professional identity formation as physician-scientists. It is important to further examine how MD-PhD-program structures, cultures, and social interactions can lead to feelings of alienation and experiences of impostor phenomenon, particularly for students from diverse and underrepresented populations in medicine.

## Background

In the 1950s, MD-PhD dual-degree training programs were created to train physician-scientists in the U.S. and strengthen the biomedical-research workforce [1]. In 2021, there were 123 U.S. medical schools offering dual-degree MD-PhD programs [2]. MD-PhD students undergo training in patient care and research, with multiple transitions between phases [3], generally completing training in 8 years [4–6]. Concordant with their intentions at graduation [7], MD-PhD students are more likely than other MD graduates to pursue an academic research career [8].

MD-PhD students and graduates comprise 2–3% of medical-school enrollees [6, 7] and graduates [9, 10]; they also are less diverse compared with other medical students and graduates [7]. A national-cohort study of MD-PhD matriculants reported MD-PhD-program attrition was 27% overall [6]. However, the 35% attrition rate for underrepresented groups in medicine (URiM), including Black/African American, Hispanic, and American Indian/Native Alaskan students, was higher than for other racial/ethnic groups (26.9% of white, 24.1% of Asian/PI, and 9.6% of other/unknown) [6]. Reasons for differing rates of MD-PhD program attrition were not examined. Studies of undergraduate college students from racial/ethnic minority groups have reported that minority-status stress, low self-esteem, and psychological distress were associated with feeling like an impostor and poor mental health [11–13]. Whether these psychosocial factors mediate higher rates of MD-PhD program attrition among URiM students is unknown [6].

Impostor phenomenon is experienced by some high-achieving, successful individuals who believe that their successes are not deserved, happening by luck and not their own competence and effort [14, 15]. Research has examined impostor phenomenon across the continuum of education, postgraduate training, and practice in medicine and other health professions [16–32]. Impostor phenomenon also has been studied among PhD students and graduates [33–37]. However, impostor phenomenon remains understudied among MD-PhD students. Impostor phenomenon among MD-PhD students may be induced by lack of demographic diversity among peers [7], a highly competitive training environment [38, 39], and sense of not belonging [40]. Implications of experiencing impostor phenomenon among students and professionals in medicine have included negative health outcomes (for example lower perceived wellness [14], greater anxiety [41] and distress [42]), high levels of burnout [18, 43, 44], self-doubt [41], and struggles with perfectionism [17].

Professional identity formation [45] as a physician-scientist may be challenging to MD-PhD students, as MD-PhD training requires development of dual identities as a physician and scientist [46], with constant tension between each identity [47]. Challenging and negative experiences during transitions between training phases can affect the process of professional identity formation due to unsatisfying socializing experiences with peers and faculty in each phase [3]. Research shows that engineering education researchers’ impostor experiences and professional identity formation may be shaped through the process of “othering” [48, 49], which marks individuals as different or not belonging to a group. The process of othering could be based on various identities, including professional and racial/ethnic, cultural and gender identities [11, 13, 50–52]. Systemic bias could promote othering by making certain individuals more vulnerable to being marginalized and experiencing impostor phenomenon. This may be especially true for women and URiM students as well as first-generation college graduates, who are underrepresented among MD-PhD physician-scientists [53]. Minoritized individuals may feel invisible, struggle to develop a sense of belonging, and experience impostor phenomenon because of their perceived outsider status [35]. This exploratory study aims to answer the question, “Why might MD-PhD trainees in the U.S., particularly those from underrepresented groups, experience IP?”

## Methods

### Setting and participants

Following IRB approval at Washington State University, business cards with a link to the study website were distributed in person at the 2017 Association of American Medical Colleges Annual Meeting to advertise the study. Eligibility criteria (noted on the study website along with a link to the survey) included being a current medical student, resident, or member of the faculty at a U.S. medical school and having experienced impostor phenomenon. For this analysis, survey respondents who were then-current U.S. MD-PhD students or residents with MD-PhD training were included.

### Design

A brief, online Qualtrics survey included demographic questions (including age, sex, race/ethnicity, name of department and university, current stage in training, and year of training for students), the validated 20-item Clance Impostor Phenomenon Scale (CIPS) [54, 55], and an open-ended question asking respondents to describe an occasion when they had experienced impostor phenomenon. At the end of the survey, respondents also were invited to complete a follow-up semi-structured interview at their convenience [56, 57].

The CIPS was used with permission and is available from Dr. Clance [54, 55]. CIPS item-response options ranged from 1 (not at all true) to 5 (very true), with higher total scores (range: 20–100) indicating more frequent/severe impostor experiences. Table 1 provides impostor-phenomenon-category scores [54]. The CIPS includes items about commonly reported impostor experiences, such as feeling like a pretender or less intelligent or competent than others are, fearing that one’s lack of competence will be discovered, and avoiding or dreading evaluations by other people.

**Table 1:** Characteristics of nine MD-PhD students and residents interviewed

Characteristics	Participants
Current stage in training	Student: 6
	3rd year: 3 (Jacob, Lily, William)
	4th year: 1 (Maya)
	5th year: 1 (Samuel)
	7th year: 1 (Lucy)
	Resident: 3 (John, Nicole, Thomas)
Age (years)	20–29: 5
	30–39: 4
Sex	Male: 5
	Female: 4
Race/ethnicity	Asian: 2
	Black: 1
	Hispanic: 1
	Native American: 1
	White: 4
Participant CIP scores^a^	Moderate (41–60): 2
	High (61–80): 3
	Intense (81–100): 4
	Range: 46–96 (out of 100)
	Mean (SD): 74.1 (16.9)

*Abbreviations*: *CIP* Clance Impostor Phenomenon, *SD* Standard deviation

^a^Each of the 20 CIP items were scored on a 5-point scale (1 = “not at all true”, 2 = “rarely”, 3 = “sometimes”, 4 = “often”, 5 = “very true”). Higher total scores indicate greater frequency and severity of IP experiences that interfere with one’s life. Scores ≤40 indicate few IP experiences, 41–60 indicate moderate IP experiences, 61–80 indicate high IP experiences, and > 80 indicate intense IP experiences ^54^

### Data collection

Open-ended interview questions were created after identifying literature gaps and finalized in consultation with MD-PhD faculty at Washington State University College of Medicine. The first author conducted all interviews, taking reflective notes during each 40–50-min interview to document her biases and decision-making. Interviews were audio-recorded and transcribed through a professional agency. Based on previous work [3], interview questions focused on programmatic experiences/challenges, socializing experiences, family and personal background, and program-transition experiences/challenges. Probing questions were asked based on responses to elicit additional/clarifying information.

### Data analysis

Transcripts were analysed using the constant comparative method [58, 59] and analytic induction [60, 61]. The constant comparative method involved analysis of each transcript and comparing subsequent interviews with each of the others, making data analysis an iterative process and interpretation both organic and evolving [58, 59]. This iterative process of inductive reasoning is commonly used in phenomenological research when researchers seek to gain detailed understanding of a particular phenomenon [60, 61], such as impostor phenomenon. Data were coded independently by two coders who each had training in use of this analytic process.

The first author led the coding process and trained a PharmD student how to code using the comparative method and inductive thematic analysis. They independently read each interview and identified key concepts, which were coded by hand with periodic input from a physician faculty member with qualitative-research experience. Guided by the research question, a list of codes emerged during open coding [59] (e.g., identity, mentoring, personal background, family, peer, transition, and stress), pointing to reasons why MD-PhD trainees may experience impostor phenomenon. Codes were constructed by both the coders, and together they developed the codebook, which was then used by each coder to independently review the transcripts once again. Coding discrepancies were discussed until a consensus was reached. For each theme that emerged from the inductive analysis, illustrative quotes are presented, with clarifying information added by the authors in brackets.

### Rigor

Transcripts were shared with the respective participants to ensure transcription accuracy; participants could add/delete/edit the text as necessary to improve accuracy/trustworthiness [62]. All authors provided input on the emerging themes, mindful of their roles and various backgrounds and perspectives, as authors’ worldviews can influence how data are collected and analysed [63].

## Results

Of 959 survey respondents in science and medicine, 13 were MD-PhD students and residents. Nine of them (six students and three residents) from seven MD-PhD programs also completed an interview. Interviewees’ characteristics and pseudonyms are shown in Table 1. Interviewees were from diverse racial/ethnic groups, and four out of nine were female. Using published CIPS cut-off scores [54, 55], the nine interviewees experienced moderate-to-intense impostor phenomenon, with scores ranging from 46 to 96. CIPS scores were similar between four survey-only respondents, who were all White women (mean: 71.8; SD: 15.2), and nine survey-and-interview respondents (mean: 74.1; SD:16.9), falling in the high- impostor-phenomenon category (Table 1). Cronbach’s alpha was 0.96, measuring the internal-consistency reliability of items on the CIPS in this sample of nine MD-PhDs.

Four interconnected themes related to MD-PhD training in each phase emerged from thematic analysis of the nine interview transcripts: professional identity formation, fear of evaluation, minority status, and program-transition experiences. Representative quotes are presented here and in Table 2.

**Table 2:** Themes linked to the impostor phenomenon among MD-PhD students

Themes	Quotes
Professional-identity formation	“We were viewed as a special group, not good enough to take care of people [as medical students] and [a] dilettante doing research, but not as much or as well as other doctoral students. I discounted my achievements with self-deprecating thoughts like, ‘I networked my way into getting accepted, I didn’t deserve this. I’m not good enough.’” (John) I wouldn’t do well because no one in my family was in medicine, and my undergraduate degree wasn’t competitive enough for me to compete in medical school. (Maya) I was always studying on my own. I did not have any support. There was a lot of social exclusion, some students work together in groups, and they never invite or reach out to other [MD-PhD] students. (William)
Fear of evaluation	“I believe that when I eventually fail, people would say, ‘Oh, he failed. He messed up, dropped the ball. This is who he truly is. He doesn’t deserve this.’” (Samuel) When you first start out, you get asked all these really basic questions, sometimes about different orders and stuff for nurses. A big part of the impostor phenomenon is questioning your ability and competence when you don’t have experience, the right answers, or the ability to say things confidently. (Thomas) I try to hide my weaknesses in the research side of things because I haven’t done as much research as others in my program. I think they [faculty] don’t realize how weak I am in that area. I think I’m weak. That could also be impostor phenomenon. (Lucy)
Minority status	“There are very few Native American physicians and physician-scientists. The health issues of Native Americans are poorly understood. I was admitted because I’m Native American and a diverse student. I am an affirmative action admit, that is why I feel like an impostor. (Maya) Sometimes, the patient questions people who are underrepresented more than other doctors, that tendency where people, based on appearances, have different expectations for them. That’s where that sense of being an impostor comes from. (Samuel) I definitely know that it [my minority status] is being considered since it helps the grant look good when it has been used to promote diversity. (Samuel)
Program-transition experiences	“During transition points, you’re rusty at whatever you’re transitioning to.” (Samuel) The transition between being in a [preclinical] classroom, having very set goals, and this is a test that you’re gonna study for. This is what’s gonna be on the test. Here’s the date. That, as opposed to being in the lab. It’s just like hey, here’s a project. Go make it work. That is very different. (Thomas) [Reintegrating into medical school was] really hard on me, because I had forgotten a lot of clinical knowledge. Sometimes I’ll hear a drug name and I’m like, ‘Oh, my God. I know that’s a common drug and everyone knows it, but I don’t remember, exactly, what it is, and I have to look it up.’ That probably, definitely, adds to the challenge. Just being rusty. (Samuel)

### Professional identity formation

All participants reported struggling with professional identity formation as MD-PhD trainees. They lacked a sense of belonging in either medicine or research, worrying that they would not have a successful clinical or research career by trying to do both. They reported being treated as outsiders by their MD-only and/or PhD-only peers and struggled to “fit in,” fearing that they will be “found out” as having no expertise in either patient care or research. Samuel felt inadequate, anxious about fulfilling dual roles, adding, “Each side undervalues the other one. Both MDs and PhDs told me that I’ll be weaker for trying to get both [degrees], focusing on two things as opposed to strengthening one.”

Feeling excluded by their MD-peers made participants who were first-generation college graduates question their feelings of competence and belongingness. Jacob helped at a volunteer clinic, did his first rounds by himself, wrote reports for the attending and passed. Yet, as a first-generation college graduate, he felt he “lacked the pedigree.” He viewed his peers from highly educated families as more competent and better prepared due to early exposures and planned entry into MD-PhD programs. He felt socially excluded. “Just being good at science isn’t always important. It’s about social skills. My peers socialized with people that are more similar to them in background, ideologies, and even looks.” Although not a monolithic group, many medical students come from well-educated, high-income families. Participants who did not share such a background felt like outsiders.

[Being a first-generation college graduate] contributes to impostor feelings, as though I don’t belong or fit in and don’t have the same know-how. (Maya)

Lucy also commented on the “culture of medicine,” where “everyone’s pretending that they know more. You should look confident around patients, even if you’re not. … I feel like everyone knows something that I don’t.”

Some participants’ peers in PhD-only programs did not want to study or conduct research with them, reinforcing participants’ feelings of not belonging. When Nicole began research training, her PhD peers already had well-defined research questions, and some completed several years of research before starting PhD training. She felt unqualified, lacking the “background, experience or papers to be at an elite institution. I was depressed. My ‘impostorness’ peaked and was related to my general well-being. I just didn’t see myself there.” Challenged to develop a researcher identity, Lily was “waiting to be uncovered,” fearing that her advisor would remove her from research papers. It was especially difficult for participants whose families did not value a science career and did not try to understand what a scientist does. Maya’s family believed she was in nursing school “working in a lab and playing with poop [she was studying parasitology],” not trying to understand her career path. Her impostor feelings stemmed from not conforming to her parents’ ideas of an appropriate career for her.

### Fear of evaluation

Seven participants shared that they feared being evaluated harshly by colleagues and patients and felt anxious about serving the dual roles expected for physician-scientists. Lucy believed that she was accepted into the program by mistake.

Oh, [I feel like an impostor] all the time. I was accepted into [university name] for the MD/PhD program and did not feel like I fit in at that school because I did not feel like I deserved to have been accepted. … I could make a mistake for four years, but eight years was a long time. … It was a mistake.

She added that she did not always have the correct answers or the ability to say things confidently when questioned. Some participants were doubly concerned that both their research- and clinical-skills would be judged poorly.

During clerkships, participants feared that they were “behind” their MD-program peers. Jacob felt incompetent and worried about his self-perceived weaknesses in clinical skills: “I get so anxious about performing poorly that I psyche myself out, and I perform more poorly. I feel anxious when someone else is relying on me, or if I am referred as an expert.” Yet, he was at ease while performing a laceration repair for a patient without “having the attendings [and] the residents there watching me.” William also described:

Being examined by clinicians or compared to other medical students, that’s pretty scary to me. Despite using a very systematic way of patient presentation, my IQ drops. I stumble and get lost on non-relevant clinical findings during evaluation, leading to internalizing that I cannot accomplish this high standard being set for me.

Some participants were intimidated by conference presentations, worrying that they would not be able to answer questions and expose their weaknesses in research training. Participants especially feared being judged as incompetent by their advisors. Lily felt anxious asking questions in lab meetings, wondering if they would be judged as “dumb questions” and her peers and advisor would laugh at her. She said, “One time I asked, ‘What is a Western blot?’ And everyone thought I was joking, but I actually didn’t know what a western blot was.”

Samuel’s insecurities stemmed from hearing his PhD peers comment that MD-PhD students, “do shortened PhDs, finishing within four years, so it’s like, ‘Oh, you’re not really doing the PhD.’ I feel more vulnerable, like an impostor.” Sometimes, these fears transcended reality. Jacob continuously feared a call from his advisor telling him, “‘I don’t know why you’re here. Someone must have fallen asleep at the job, because you need to show yourself the door.’ I have screaming doubt at whatever I do.”

### Minority status

Five participants wondered if their minority status gave them preferential treatment for program acceptance and felt particularly alienated, especially if they heard comments from their peers to that effect. Samuel perceived that patients were sceptical about being treated by him and sometimes responded to him differently than they did to his White peers. He second-guessed himself when questioned by patients about their disease or prognosis. Other participants wondered if they received special consideration in the competition for MD-PhD program acceptance due to their minority status, worrying that whatever they did or said would be considered representative of all minorities from their background.

Maya spoke about being a first-generation college graduate from a relatively poor family. She talked about the dualism in her understanding of health through her family and through her experience of Western medicine, all of which contributed to her impostor phenomenon. John felt his non-White status led to being scrutinized more harshly and to having his comments dismissed and his clinical decisions criticized more than White students’ decisions. “When you start getting more micro-aggressions, you wonder whether you are in the right place. All of these things contribute to that [IP].”

Some participants experienced impostor phenomenon whenever they were selected for scientific awards. Samuel’s peers often commented how his diversity status had helped him win scholarships and awards, because the program wants to promote diversity. Such comments singled him out and made him feel unworthy of these awards. “People treat you like an impostor, like, ‘Maybe you’re gonna get it [an award] ‘cause you’re a minority.’” John was told, “You’re the face of the program because you’re a minority.” He was often the only person from a minority group at conferences, “feeling like I was the odd person out. I thought I would be questioned because of that.”

### Program-transition experiences

Six participants reported considerable difficulties transitioning between training phases and integrating into the cultures of medicine and/or research, feeling anxious for not assimilating well in either culture. Participants constantly played catch-up, both socially and culturally, transitioning between preclinical training and research and then again between research and the start of clinical training.

Transitioning from a structured, preclinical training (medical school) into a less-structured research training (graduate school) was anxiety provoking. Thomas shared, “There were times where I did not remember all these minutiae [about conducting experiments].” Participants did not always have resources for quickly learning scientific-writing skills. PhD advisors were generally not clinically trained, lacking clinical-research expertise to provide necessary guidance. Nicole commented, “I didn’t know where I was going with my research, partly because I wasn’t quite sure what my clinical focus would be.” A shorter PhD timeframe (4–5 years) meant some students were not able to repeat experiments once medical training restarted or to publish findings in a timely fashion due to the lengthy manuscript review and revision cycle. Nicole also doubted if she could pursue a research career successfully, because her research interests were “more patient-focused than the basic science [gene] signalling research that I was doing for my PhD.”

Re-entering medical school to begin clinical clerkships after several years away from medicine also was challenging. Re-entering students were now completing clerkships with much younger third-year medical students who had not taken a break to complete PhD training. John felt like “an expert of this little sliver of science” with his medical knowledge rusty and inadequate. “That transition was very hard for me. … I wasn’t as fresh and didn’t retain a lot of what I’d learned. I didn’t belong there.” Nicole struggled with the emphasis on clinical trials and statistics related to those clinical trials. “I didn’t have a whole lot of stats training.” Samuel did not have an MD-PhD mentor, and his PhD mentor “couldn’t advise me much about the clinical side. I felt very stressed and uncomfortable waiting for the truth to be exposed, that sensation of dread that I had was heightened.”

## Discussion

Four interconnected themes emerged from the analysis describing challenges that MD-PhD participants attributed to their experiences of impostor phenomenon during both, the MD-phase and the PhD-phase of training. These themes included challenges with professional identity formation as a physician and scientist, fear of evaluation, minority-status stress, and navigating MD-PhD program transitions and acclimating to the different cultures of science and medicine. Each theme is discussed in the context of the literature.

### Professional identity formation

Among resident physicians, professional identity formation has been described as an interplay between “doing” and “being,” and is influenced by one’s personal identity and socializing experiences during residency training [64]. In our study, impostor experiences were reported to be related to challenges in identity construction as a physician and scientist. Medicine and research were viewed as culturally different. Participants felt like outsiders during each phase of MD-PhD training, unable to integrate socially in either culture. Professional identity construction for trainees interacting in these distinct cultural worlds of medicine and research, with strikingly different professional expectations, requires having role models in both worlds [64]. MD-PhD programs could foster physician-scientist identity formation through synergistic, curricular- and social-integration activities, role modelling, and mentoring.

### Fear of evaluation

Being questioned or observed at school/work [25, 65] and being judged/evaluated or ridiculed by patients, physicians, and peers could trigger impostor experiences. Participants in this study compared themselves unfavourably with others, focusing on other’s strengths and one’s own perceived weaknesses. As a characteristic of impostor phenomenon related to perfectionism [17], fear of making mistakes [66] and medical errors [67, 68], participants viewed evaluations negatively and not as constructive feedback [69]. Participants internalized lack of belonging as being excluded due to their inability to compete with peers. Feeling unable to fully assimilate into either culture of medicine or research and worrying whether they had adequately developed skills/competencies required to perform successfully in both cultures, participants compared themselves negatively with fellow students pursuing only PhD and MD degrees.

### Minority status

Participants from minority groups were acutely aware of how their demographic characteristics shaped impostor phenomenon experiences. Five participants described how they did not fit the stereotypical image of a white, male physician or scientist due to their racial/ethnic background or being a first-generation college graduate. They did not know what to expect during MD-PhD training and had little support from parents, family, mentors, and non-academic social networks, which led to feelings of alienation. Feelings of alienation due to othering [48] was profound among our participants, especially for first-generation college graduates and individuals from backgrounds historically underrepresented in medicine. The conflict between dual identities as physician and scientist could be especially seen when one’s identity as a physician or scientist (or both) is invalidated by othering through words and actions. Resident trainees from URiM groups have reported facing additional challenges with racism, bias, micro-aggressions, and sense of “otherness” [70], which also can lead to stress and anxiety associated with impostor phenomenon. Moreover, a study of first-year medical students found that some students had surprisingly high levels of impostor phenomenon before matriculation; this study also showed that impostor phenomenon increases over the first year of medical school and that higher impostor phenomenon was associated with higher neuroticism/anxiety and loneliness, and lower self-esteem and sociability [71]. Thus, given the toll that minority-status stress has on mental health outcomes among college students, medical students and residents [11–13, 35, 65, 71], experiencing the additional burden of impostor phenomenon that challenges URiM MD-PhD trainees to continue and thrive on their path to becoming a physician-scientist is a daunting obstacle to success.

MD-PhD mentors from URiM backgrounds are lacking, but if available, could provide individualized support and professional guidance to help URiM MD-PhD students navigate challenges during training [3, 72]. When URiM mentors are not available, purposefully designed closed-mentoring triads [73] could be beneficial; closed-mentoring triads would include an MD-PhD student, an MD-PhD graduate, and a faculty mentor working together and interacting closely. Such closed-triads have shown significant benefits in college students over other mentoring structures in terms of greater gains in thinking and working like a scientist, research self-efficacy, and satisfaction with research, even after controlling for students’ gender, race/ethnicity, first-generation status, prior academic achievement, and length of research experience [73]. As professional identity formation as a confident and competent physician-scientist is crucial to MD-PhD students’ retention in biomedical-research workforce, closed-mentoring triads could be crucial for MD-PhD and other students who are affected by impostor phenomenon. To our knowledge, closed-mentoring triads have not been studied as a means to mitigate the impact of impostor phenomenon on professional identity formation among MD-PhD students.

### Program-transition experiences

The purpose of MD-PhD training is to build a physician-scientist workforce with expertise in both clinical care and biomedical research [1, 4, 74]. However, MD-PhD students experience multiple transitions between phases and transition-related challenges. Such challenges include lack of MD-PhD mentors, acclimating to different, phase-specific cultures, and poor curricular integration between phases [3]. Our participants reported similar transition challenges. Peers [75] and mentors [76] were not always viewed as friendly or supportive. Transitions are dynamic and potentially stressful, requiring coping strategies and support that allow persons to function competently under new circumstances [70, 77–79]. Transitions can make individuals feel vulnerable and have implications for professional development and the provision of optimal patient care [80]. MD-PhD mentors are needed to support MD-PhD students to successfully navigate these challenges [3, 81]. Here, too, closed-mentoring triads could be helpful in creating a support network for trainees experiencing stressful transitions and impostor phenomenon. Impostor phenomenon can be addressed, managed, and reduced through inclusive learning spaces that destigmatize and normalize conversations around fear of being ousted as a fraud. In general, new medical-school matriculants may be prone to experiencing self-doubt, low self-esteem, and impostor phenomenon [30, 71], which could result in psychological distress and burnout, especially when individuals perceive they are at-risk of being exposed as fraud [30].

It should be noted that each of the four themes constructed were intertwined through the perception of othering of the self as different from the larger group of MD-PhD trainees, as described above. As seen in prior research [48, 82], persons experiencing impostor phenomenon might consider themselves as outsiders, which, in this study, was based on various identities that participants held salient. These identities might include MD-PhD trainees’ URiM, gender, or first-generation college-graduate status, and/or their struggling professional identity formation as a physician-scientist, which differs from either a physician identity or a scientist identity alone. Ultimately, preventative strategies and early interventions may be most effective when implemented at both individual and systemic levels, if possible, keeping these nuances and complexities in mind [30]. Public discussions should start early during orientation to promote cultural awareness and sensitivity [83] and greater awareness about impostor phenomenon and its associated behavioural traits [84]. Periodic lunch seminars, especially in the first few years when students are still adjusting to the program, could help MD-PhD students create their community and build a supportive local network of peers and mentors. Systemic interventions might include re-immersion programs focussed on strengthening clinical and research skills that promote smoother transitions between the different phases of MD-PhD training [3, 85], and efforts to create opportunities to engage in national networks [10] and leadership-training workshops [86]. Other strategies include facilitated peer collaborations to learn from each other and reduce isolation as well as targeted recruitment of MD-PhD students from diverse backgrounds [87]. Overall, interventions to prevent or manage impostor phenomenon could occur at both the individual and institutional level multiple times over the course of MD-PhD training.

This was an exploratory, qualitative study of impostor phenomenon in MD-PhD trainees, limited by a small sample and cross-sectional design. Although similar challenges and experiences have been reported [3], these findings may not be generalizable to the larger cadre of MD-PhD trainees. Trainees who do not experience impostor phenomenon could face similar challenges in their professional identity formation and transitions between training phases, but may have adequate mentoring/support to mitigate onset of impostor phenomenon. Future longitudinal studies should examine if impostor phenomenon in MD-PhD students evolves over time along the educational and professional continuum, why some MD-PhD trainees experience impostor phenomenon and others do not, and evaluate the effects of interventions, such as culturally sensitive mentorship on impostor phenomenon and trainee success.

## Conclusion

This is one of the first studies to examine impostor phenomenon among MD-PhD trainees. Participants identified social and situational circumstances that elicited feelings of impostor phenomenon, some describing negative effects of impostor phenomenon on their mental health. Targeted institutional and programmatic interventions could mitigate the effects of impostor phenomenon on MD-PhD trainees’ professional identity formation, feelings of alienation (especially for URiM trainees), and inability to function competently and confidently as physician-scientists. Adequate support and mentoring are essential, not only to increase the size and diversity of the MD-PhD physician-scientist workforce but also to promote their future success in improving the health of an increasingly diverse nation.

## Data Availability

Not available. The interviews generated and/or analysed during the current study are not publicly available. The interviews are confidential and contain sensitive information that may identify the participants. The consent form that participants signed stated that their data will not be shared with members outside the research group. Relevant parts of the interview could be made available from the corresponding author on reasonable request in writing after removing all identifiable information.

## Abbreviations

IP: Impostor phenomenon
CIPS: Clance Impostor Phenomenon Scale
URiM: Underrepresented groups in medicine
STEM: Science, Technology, Engineering, and Mathematics
